# Rapid Conductometric Detection of SARS‐CoV‐2 Proteins and Its Variants Using Molecularly Imprinted Polymer Nanoparticles

**DOI:** 10.1002/admt.202200965

**Published:** 2022-12-04

**Authors:** Ganganath S. Perera, Md. Ataur Rahman, April Blazevski, Alasdair Wood, Sumeet Walia, Madhu Bhaskaran, Sharath Sriram

**Affiliations:** ^1^ Functional Materials and Microsystems Research Group and the Micro Nano Research Facility RMIT University Melbourne VIC 3001 Australia; ^2^ Soterius Pty Ltd. Melbourne Australia

**Keywords:** conductometric biosensor, COVID‐19 variants, FHA, MIPs, RBD

## Abstract

Severe acute respiratory syndrome coronavirus 2 (SARS‐CoV‐2) biosensors have captured more attention than the conventional methodologies for SARS‐CoV‐2 detection due to having cost‐effective platforms and fast detection. However, these reported SARS‐CoV‐2 biosensors suffer from drawbacks including issues in detection sensitivity, degradation of biomaterials on the sensor's surface, and incapability to reuse the biosensors. To overcome these shortcomings, molecularly imprinted polymer nanoparticles (nanoMIPs) incorporated conductometric biosensor for highly accurate, rapid, and selective detection of two model SARS‐CoV‐2 proteins: (i) receptor binding domain (RBD) of the spike (*S*) glycoprotein and (ii) full length trimeric spike protein are introduced. In addition, these biosensors successfully responded to several other SARS‐CoV‐2 RBD spike protein variants including Alpha, Beta, Gamma, and Delta. Our conductometric biosensor selectively detects the two model proteins and SARS‐CoV‐2 RBD spike protein variant samples in real‐time with sensitivity to a detection limit of 7 pg mL^–1^ within 10 min of sample incubation. A battery‐free, wireless near‐field communication (NFC) interface is incorporated with the biosensor for fast and contactless detection of SARS‐CoV‐2 variants. The smartphone enabled real‐time detection and on‐screen rapid result for SARS‐CoV‐2 variants can curve the outbreak due to its ability to alert the user to infection in real time.

## Graphical abstract

Sensitive detection of severe acute respiratory syndrome coronavirus 2 (SARS‐CoV‐2) proteins is important for early identification of COVID‐19 infection. The conventional SARS‐CoV‐2 biosensors often produce false results due to poor sensitivity and sensor degradation. Novel high resistivity silicon‐based conductometric sensor modified with SARS‐CoV‐2 molecularly imprinted polymers for rapid, sensitive, selective, stable, and accurate detection of SARS‐CoV‐2 proteins in human saliva is introduced.

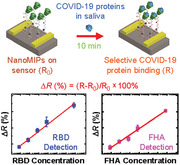

## 1 Introduction

With the outbreak of the COVID‐19 global pandemic in early 2020, an immense scientific effort was undertaken to explore different severe acute respiratory syndrome coronavirus 2 (SARS‐CoV‐2) virus detection techniques. To date, a wide range of such detection methods have been developed from lab‐specific techniques to point‐of‐care (POC) devices.^[^ ^1^, ^2^, ^3^, ^4^, ^5^ ^]^ Conventional diagnostic methods for the detection of SARS‐CoV‐2 virus can be classified into three primary categories: i) computed tomography (CT) scan, ii) nucleic acid‐based tests, and iii) serological immunoassay‐based tests. Although CT scans are capable of identifying COVID‐19 infection and the severity of the disease, CT systems suffer from drawbacks such as expensive instrumentation, the requirement of expert skills, low specificity (25%), and imposing biological impacts due to X‐ray radiation exposure.^[^ ^6^, ^7^ ^]^

Among all reported SARS‐CoV‐2 detection methods, nucleic acid‐based tests are the most widely used across the globe.^[^ ^8^, ^9^ ^]^ Sequencing methods (Sanger and next‐generation sequencing (NGS)),^[^ ^10^, ^11^ ^]^ reverse transcription‐polymerase chain reaction (RT‐PCR),^[^ ^12^, ^13^ ^]^ loop‐mediated isothermal amplification (LAMP),^[^ ^14^, ^15^ ^]^ and clustered regularly interspaced short palindromic repeats (CRISPR)^[^ ^16^, ^17^ ^]^ are among such different nucleic acid‐based tests. RT‐PCR is considered as the gold standard in COVID‐19 diagnosis and sequencing methods (Sanger and NGS) are important in both diagnosing COVID‐19 infection and evaluating its genomic mutations. Despite being the most popular SARS‐CoV‐2 detection methods, these conventional nucleic acid‐based tests also undergo several shortcomings including the generation of false positive/negative results, long detection time (>2 h), expensive instrumentation, and complex sample preparation.^[^ ^13^, ^18^ ^]^ To compensate the shortcomings of nucleic acid‐based tests, serological immunoassay‐based techniques have been introduced which are cost‐effective methods and rapid diagnostics of the SARS‐CoV‐2 virus.^[^ ^19^, ^20^ ^]^ Traditionally, lateral flow immunoassay (LFA)^[^ ^21^ ^]^ and enzyme‐linked immunosorbent assay (ELISA)^[^ ^22^ ^]^ are used as two main immunoassay‐based COVID‐19 diagnostic methods. However, the requirement of high viral/antigen load, wide range of responses causing the low sensitivity and low specificity of these techniques lead to false positive/negative results, the degradation of biological components in the system (e.g., enzymes in ELISA, and declining levels of antibodies over time), and the requirement of molecular tags for the detection (e.g., fluorescence and luminescence) are some of the noticeable challenges of serological immunoassay‐based methods.

Alternatives to the aforementioned conventional COVID‐19 diagnostic techniques include an array of biosensors that fulfill the ASSURED criteria (affordable, sensitive, specific, user‐friendly, rapid and robust, equipment‐free, and deliverable to end‐users) prescribed for a biosensor by the World Health Organization (WHO).^[^ ^23^ ^]^ Based on the signal sensing method and transduction principles, the reported SARS‐CoV‐2 biosensors can be classified into i) optical^[^ ^24^ ^]^ (e.g., surface plasmon resonance,^[^ ^25^ ^]^ fluorescence resonance energy transfer (FRET),^[^ ^26^ ^]^ surface‐enhanced Raman spectroscopy (SERS),^[^ ^27^ ^]^ and fiber optics^[^ ^28^ ^]^), ii) colorimetric,^[^ ^29^, ^30^ ^]^ iii) electrochemical,^[^ ^31^, ^32^ ^]^ iv) piezoelectric,^[^ ^33^, ^34^ ^]^ v) magnetoelastic,^[^ ^35^ ^]^ vi) field‐effect transistor (FET),^[^ ^36^, ^37^ ^]^ and vii) microfluidic^[^ ^38^ ^]^ biosensors. The key underlying operational principle of these biosensors is the generation of a detectable signal due to the interaction of a specific bioreceptor in the biosensor with the incoming SARS‐CoV‐2 component such as the receptor binding domain (RBD) of the spike (*S*) glycoprotein. The prevailing molecular interaction in most of these biosensors is the antibody–antigen interaction. The sensor surface is modified with SARS‐CoV‐2‐specific antibodies and the signal is produced upon interaction with SARS‐CoV‐2 antigens. Although the above‐mentioned SARS‐CoV‐2 biosensors are embedded with their unique advantages and disadvantages in detection techniques, one of the key and common challenges of these biosensors to compete against the conventional COVID‐19 diagnostic tools is the low sensitivity and low specificity of the measurements leading to false positive/negative outcomes. This occurs primarily due to the biological antibodies that are immobilized onto the sensor surface degrading with time causing the gradual decrease in antigen detection efficiency.

Molecularly imprinted polymers (MIPs) are synthetic antibodies that are a great source to overcome this above‐mentioned challenge. MIPs are physically and chemically stable in a wide range of conditions, and highly specific for the antigen they are designed to bind. Biosensing applications using MIPs have been reported before.^[^ ^39^, ^40^, ^41^ ^]^ Recently, the utility of MIPs as SARS‐CoV‐2 biosensors has been in discussion^[^ ^42^, ^43^, ^44^ ^]^ and several studies were reported on their actual usage.^[^ ^45^, ^46^ ^]^ As an example, Raziq et al. developed a MIP‐based electrochemical sensor for detection of SARS‐CoV‐2 nucleoprotein for a nasopharyngeal swab sample.^[^ ^45^ ^]^ Furthermore, Cennamo et al. performed a proof‐of‐concept study on a surface plasmon resonance‐plastic optical fiber (SPR‐POF) sensor for detecting SARS‐CoV‐2 *S* protein (S1 subunit specifically) at a detection limit of 58 × 10^–9^ m.^[^ ^46^ ^]^ In multiple review articles, Ramanavičius et al. have reported the capability of using polypyrrole as an imprintable conducting polymer for MIP‐based electrochemical biosensors.^[^ ^47^, ^48^, ^49^ ^]^ In one of their latest studies, they have demonstrated that molecularly imprinted polypyrrole deposited Pt electrodes could successfully detect SARS‐CoV‐2 spike glycoprotein with a detection limit of 5 µg mL^–1^.^[^ ^50^ ^]^ In this study, pulsed amperometric detection was employed to detect the SARS‐CoV‐2 spike glycoprotein and 2.1 times higher change of current was observed for molecularly imprinted polypyrrole modified electrode compared to polypyrrole without imprinted electrodes. In addition, they also studied the capability of detecting recombinant SARS‐CoV‐2 spike protein antibodies by using recombinant SARS‐CoV‐2 spike protein immobilized premodified gold electrodes where the detection limits of 2.53 × 10^–9^ and 1.99 × 10^–9^ m for recombinant SARS‐CoV‐2 spike protein antibodies were observed under cyclic voltammetry and electrochemical impedance spectroscopy, respectively.^[^ ^51^ ^]^ Given the promising potential of MIPs as SARS‐CoV‐2 biosensors, more research is required to be conducted.

In the current study, we have introduced a novel MIP‐incorporated conductometric microbiosensor for sensitive, selective, and rapid detection of SARS‐CoV‐2 proteins and its variants in artificial saliva and phosphate buffered saline (PBS). This two‐terminal electrode biosensor is photolithographically fabricated on a commercially available intrinsic silicon wafer. Conductometric biosensors^[^ ^52^, ^53^, ^54^ ^]^ and silicon wafer‐based biosensors^[^ ^55^, ^56^ ^]^ have been reported before. However, MIP‐incorporated silicon wafer‐based conductometric biosensors for SARS‐CoV‐2 protein detection have not been reported before, to the best of our knowledge. The current study reveals that careful selection of intrinsic silicon wafers with suitable resistivity is useful in SARS‐CoV‐2 protein detection. Using commercially available intrinsic silicon wafers in biosensing applications have several advantages including easy fabrication of the sensing device, no requirement of extra sensing layers, ability to customize different geometric designs of the device with minimal material wastage and cost effective. In this study, RBD of spike (*S*) glycoprotein and full‐length trimeric spike protein have primarily been used as the model target SARS‐CoV‐2 proteins along with several other commercially available SARS‐CoV‐2 RBD protein variants (Alpha, Beta, Gamma, and Delta strains). For the sake of easiness in interpreting the protein name, RBD of spike (*S*) glycoprotein will be denoted as RBD protein and full‐length trimeric protein will be denoted as FHA protein (considering the modifications to the spike sequences to stabilize it as a trimer) from here onwards in the manuscript. Electrical signals (i.e., resistance) were obtained before the SARS‐CoV‐2 protein addition and after SARS‐CoV‐2 protein addition. The underlying mechanism for the change in the resistance values upon addition of SARS‐CoV‐2 proteins was determined to be via the changes in the surface charge transfer occurring due to the intermolecular interactions of incoming proteins with the binding sites of MIPs. Highly selective and sensitive detection of RBD and FHA proteins was observed in real‐time monitoring. Capability of sensitive and selective detection of these two types of SARS‐CoV‐2 proteins ensures that the results from our biosensors are accurate (no false negative/positive outcomes in COVID‐19 diagnosing). Our biosensor was also capable of rapid identification of different SARS‐CoV‐2 RBD protein variants used in this study in both PBS and artificial saliva. Another key finding from this study is that these biosensors can be reused for the detection of RBD proteins for at least one reuse cycle by treatment with pure isopropyl alcohol (IPA). We also developed a mobile app where the results from the biosensor can be wirelessly transferred to a smartphone via near‐field transmission (NFC) in less than 10 s. Due to the simple and miniaturized sensor geometry and relatively straightforward detection technique employed, our biosensor is compatible to integrate with the conventional portable electronics and wearable devices. It is important to note that the usage of this sensor platform is not only limited to SARS‐CoV‐2 protein detection. By careful selection of biomaterials to functionalize the device surface, this platform can be further extended to detect an array of biomolecules including DNA, antigens, and other viral sub‐types. Hence, this proposed silicon‐based conductometric biosensor platform will play an important role for a new category of point‐of‐care medical devices in the future.

## 2 Results

### 2.1 Conductometric Biosensor in SARS‐CoV‐2 Protein Detection

**Figure 1:**
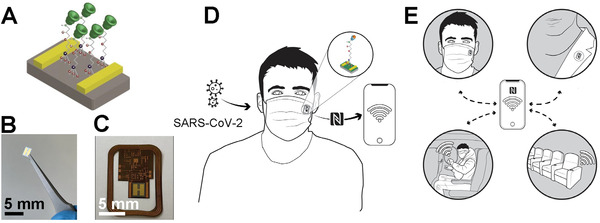
Summary of SARS‐CoV‐2 biosensor and its applications. A) The schematic representation of the COVID‐19 nanoMIPs‐immobilized silicon device platform. B) Optical image of the SARS‐CoV‐2 biosensor. C) Optical image of the SARS‐CoV‐2 biosensor integrated into the NFC flexible circuit. D) Depiction of SARS‐CoV‐2 virus detection from the SARS‐CoV‐2 biosensor attached to a face mask and NFC data transmission. E) Potential applications of the SARS‐CoV‐2 biosensor.

**Figure 2:**
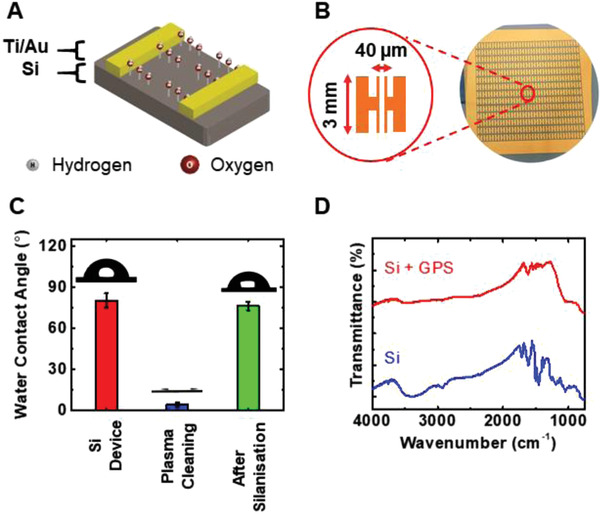
SARS‐CoV‐2 biosensor fabrication and characterization. A) The schematic representation of silicon device platform. B) Optical image of device arrays. The magnified image shows the geometry of an electrode pair of the electrode arrays. C) Water contact angle measurements for freshly fabricated silicon device, after O_2_ plasma cleaning, and after GPS silanization. D) FTIR spectra of silicon devices before and after GPS silanization.

The operational principle of the biosensor in SARS‐CoV‐2 protein detection and its potential applications is summarized in **Figure** **1**. Figure 1A depicts the summary of the functional biosensor which consists of COVID‐19 nanoMIPs bound to the GPS silanized two‐terminal electrical device. The details of the components of the biosensor are described later in **Figure** **2**A. Figure 1B displays the optical image of the miniaturized biosensor. Figure 1C demonstrates the integration of the biosensor into the NFC flexible circuitry for the wireless transmission of data to the mobile app. The details of the associated electronics will be discussed later in the manuscript. Figure 1D depicts the potential insertion of the biosensor integrated circuitry to a face mask as a wearable sensor and the wireless data transmission via NFC to the mobile app. Figure 1E summarizes the potential user case scenarios where this COVID‐19 sensor can be important such as in personal protective equipment (PPE), wearable device, public transportation, and public event venues.

### 2.2 Characterization of the Biosensor

Figure 2A shows the cross‐section of the device platform. As shown in the image, this device primarily consists of photolithographically fabricated Ti/Au two‐terminal electrodes on the high resistivity silicon wafer (>5000 Ω cm). Freshly fabricated device surface is always associated with dangling hydroxyl groups which are important in the following steps of silanization. Figure 2B demonstrates the geometry of the two electrodes patterned on the silicon wafer. The corresponding geometry was selected to facilitate the placement of the resistance measuring probes. A series of resistance measurements were obtained at different electrode gaps (Figure S1A, Supporting Information). Considering the packing density of the nanoMIPs and the optimized baseline resistance values, the electrode gap was selected to be 40 µm for the biosensor. Furthermore, optimization of the channel lengths was conducted by changing the length of the electrodes (2, 3, and 4 mm in length) (Figure S1B, Supporting Information). The resistance values for across the three lengths did not show any significant changes. Henceforth we considered the sensing areas in determining the optimum channel length. At 2 mm channel length, nanoMIP packing is insufficient for a high signal response due to the lower sensing area. At 4 mm channel length, the nanoMIP packing is increased, but there could be a drop of electrical signal due to the long charge transfer pathway. This hypothesis was tested based on the RBD detection at different channel lengths (Figure S2, Supporting Information). The highest resistance change for as‐received RBD protein was obtained for the electrodes with 3 mm long channel length. Based on these results, 3 mm channel length was selected as the optimum length of the electrodes.

The device surface was then functionalized with GPS silane (Figure 2C). In our recent publication on oxygen‐deficient ZnO conductometric biosensors in detecting inflammatory biomarkers, the device surface was silanized with GPS to ensure the immobilized antibodies were orientated in an ordered manner to maximize binding of incoming antigens.^[^ ^52^ ^]^ Henceforth, we functionalized the silicon device surface with GPS silane for ordered binding of COVID‐19 nanoMIPs to the device. GPS silanization was confirmed based on water contact angle measurements (Figure 2C) and Fourier transform infrared (FTIR) spectroscopy (Figure 2D). The water contact angle of the freshly fabricated silicon device was 80.2 ± 5.1°. Prior to the GPS silanization, the device surface was O_2_ plasma cleaned to remove any organic contaminants and to activate the hydroxyl groups on the surface to facilitate the GPS binding. After O_2_ plasma cleaning, the surface became extremely hydrophilic with a water contact angle of 4.2 ± 1.4° and similar results were observed in previous studies.^[^ ^52^ ^]^ After the devices were GPS silanized, the water contact angle increased to 76.1 ± 3.2° indicating a modification to the system after GPS vapor exposure. The device surface became extremely hydrophobic after GPS silanization with respect to the O_2_ plasma cleaned surface due to the long carbon chain in GPS molecule.

The FTIR spectra depicted in Figure 2D further confirm the successful GPS silanization. FTIR spectral features of a freshly fabricated silicon device have clearly modified after exposing the devices to GPS silane suggesting that GPS is bound to the silicon device. The broad peak at ≈3500 cm^−1^ and multiple peaks between 1000 and 1500 cm^−1^ region for the silicon device are attributed to the OH stretching vibration band of adsorbed water molecules and Si—O stretching vibration modes of oxygen impurities, respectively.^[^ ^57^, ^58^ ^]^ Once the silicon device was exposed to oxygen plasma followed by GPS silanized, a FTIR spectrum with much smooth baseline was observed indicating the oxygen plasma has cleaned the silicon surface successfully. The peaks at ≈1000 cm^−1^ and 788 cm^−1^ in the FTIR spectra of GPS silanized silicon devices are assigned to Si—O—Si asymmetrical stretching and Si—O stretching vibrational bands of GPS on silicon device, respectively.

Further evidence supporting successful GPS silanization was provided by the resistance measurements obtained for the silicon device before and after GPS silanization (Figure S3A,B, Supporting Information). The resistance value for the freshly fabricated silicon device had dropped ≈70% after GPS silanization suggesting the silicon surface interacted with the GPS silane.

### 2.3 Detection of RBD and FHA Proteins

**Figure 3:**
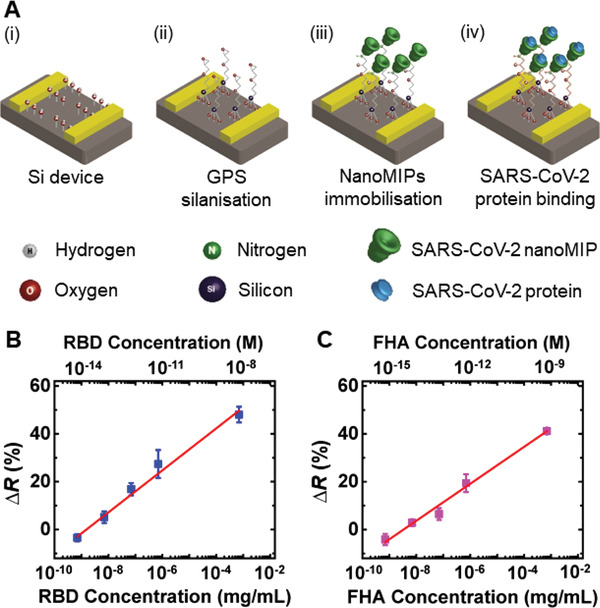
Schematics of SARS‐CoV‐2 protein binding and change in resistance values for two SARS‐CoV‐2 proteins. A) The schematic representation of the stepwise development of the SARS‐CoV‐2 biosensor. B) Change in resistance as a function of RBD concentration. C) Change in resistance as a function of FHA concentration. The nominal concentration of COVID‐19 nanoMIPs is 0.339 mg mL^−1^. The sample size (*n*) is 5 for the statistical analysis of each data point.

Stepwise development of the SARS‐CoV‐2 biosensor is depicted in **Figure** **3**A. The freshly prepared silicon device surface is rich in dangling hydroxyl groups (Figure 3A‐i). O_2_ plasma cleaning for the freshly fabricated device activates the hydroxyl groups on the device surface. Once the device is exposed to GPS silane, the hydroxyl groups on the device surface interact with the GPS silane and form Si—O bonds (Figure 3A‐ii). The epoxy ring of the GPS silane is then reacted with the incoming COVID‐19 nanoMIPs to facilitate the ordered binding of the nanoMIPs (Figure 3A‐iii). The COVID‐19 nanoMIP binding to GPS silanized devices was characterized via two methods, i) FTIR spectra (Figure S4A, Supporting Information) and ii) resistance measurements (Figure S4B, Supporting Information). The significant change in the FTIR spectral features of the GPS silanized devices in the presence of nanoMIPs certainly suggests that the GPS had interacted with the COVID‐19 nanoMIPs (Figure S4A, Supporting Information). Such a drastic change in FTIR spectral features of GPS silanized devices was not observed when treating with PBS which was utilized as a negative control. Upon addition of COVID‐19 nanoMIPs to the GPS silanized devices, the resistance drops only by an average of 25% whereas for the PBS control the resistance drops by an average of 78% (Figure S4B, Supporting Information). Such a drastic drop in resistance in the presence of PBS is likely due to the high ionic composition of the system which can be physically adsorbed to the GPS causing an increase in conductivity across the electrodes after PBS addition. Such a large change in ionic composition is not expected for nanoMIPs and the drop in resistance indicates that COVID‐19 nanoMIPs act as electron donors upon GPS interaction, thus contributing electrons to the system causing a reduction in resistance. In the final step, the COVID‐19 nanoMIP immobilized‐biosensor binds with the incoming SARS‐CoV‐2 proteins (Figure 3A‐iv). The binding sites of the COVID‐19 nanoMIPs can selectively bind with the incoming proteins. Further evidence for COVID‐19 nanoMIPs binding with SARS‐CoV‐2 proteins is revealed by FTIR spectra (Figure S5A,B, Supporting Information). The drastic changes in FTIR spectral features of the pristine protein samples after interacting with nanoMIPs confirm the protein interaction with that COVID‐19 nanoMIPs.

Both RBD and FHA proteins demonstrated an increased change in resistance with respect to the baseline as a function of protein concentration (Figure 3B,C). The baseline resistance is the resistance of the COVID‐19 nanoMIPs immobilized GPS silanized device before the protein addition. The baseline resistance was measured to exclude the variations in the resistance across GPS silanized devices. The percentage resistance change (∆*R*%) was calculated by using the equation

(1) $$\begin{array}{c} \Delta R\left(\%\right)=\left(R-R_{0}\right){\text{/R}}_{0}\times 100\% \end{array}$$

where *R*_0_ is the baseline resistance and *R* is the resistance after protein addition.

The as‐received COVID‐19 nanoMIPs were used for the experiment without any dilution. Considering the size of the COVID‐19 nanoMIP particle size (range ≈70–150 nm), the amount of undiluted COVID‐19 nanoMIP solution (10 µL of 0.339 mg mL^−1^ nanoMIPs)) is sufficient to completely occupy the GPS molecules in the sensing area of the device and no exposed GPS molecules are available to directly interact with the incoming SARS‐CoV‐2 proteins. Considering the device surface was washed thoroughly with PBS solution after COVID‐19 nanoMIP immobilization and COVID‐19 nanoMIPs were readily soluble in PBS, it was reasonable to declare that only the COVID‐19 nanoMIPs chemically adsorbed onto the GPS molecules were retained on the surface whereas loosely bound COVID‐19 nanoMIPs were removed during the wash step of device preparation.

Both RBD and FHA proteins displayed a linear correlation for the change in resistance as a function of protein concentration (Figure 3B,C). The responsivity (i.e., the slope of the graph) for RBD and FHA proteins are 8.8 and 7.8 % mg mL^−1^, respectively. This suggests that the biosensor is more sensitive in detecting RBD protein than FHA protein. This could probably be due to the relatively small size of the RBD protein (≈30 kDa) compared to the FHA protein (≈170–180 kDa) which leads to relatively efficient charge transfer between MIP and short length of RBD protein. To evaluate the contribution from the matrix in the protein solution in resistance change, control experiments were conducted for PBS solution. The average resistance change for PBS was ‐12% which is in a reversed polarity to the change in resistance observed for both SARS‐CoV‐2 proteins, suggesting that there is no significant contribution from PBS to the resistance changes in both SARS‐CoV‐2 proteins. Therefore, the entirely of the resistance change in the SARS‐CoV‐2 protein conditions was attributed to the binding of the protein to the biosensors, without interference from the matrix.

The detection limit for both RBD and FHA proteins was determined to be 7 pg mL^−1^ as this was the concentration in which a positive change in resistance following protein addition was no longer observed. This value is more than 100 times lower in concentration than the reported clinically relevant SARS‐CoV‐2 protein concentration of 1 ng mL^−1^.^[^ ^59^ ^]^ This signifies that our simple device platform is a powerful tool for detecting SARS‐CoV‐2 proteins. As both RBD and FHA SARS‐CoV‐2 surface proteins can be detected at low concentrations in this study, our biosensors are useful in producing more accurate (no false positive/negative outcomes) and sensitive results in determining whether the user has been exposed to SARS‐CoV‐2 virus.

The shelf‐life of the GPS silanized‐high resistivity silicon devices was evaluated based on the resistance changes observed for both RBD and FHA proteins on devices with different timescales after GPS sanitation (Figure S6, Supporting Information). The nanoMIPs were freshly immobilized on these devices with different GPS silanization timescales to evaluate the device performance on RBD and FHA protein detection. Irrespective of the timescales after GPS silanization, both proteins displayed linear change for change in resistance as a function of protein concentration. The responsivity for RBD proteins was determined to be 8.8, 8.7, 8.2, and 7.9%/mg mL^−1^ for fresh, 1 month old, 2 months old, and 5 months old devices, respectively (Figure S6A, Supporting Information). These correspond to 1%, 7%, and 10% change in responsivity for RBD proteins for 1, 2, and 5 month old devices, respectively, compared to the responsivity of the fresh devices. Similarly, the responsivity for FHA proteins was determined to be 7.8, 7.6, 7.0, and 6.4%/mg mL^−1^ for fresh, 1 month old, 2 months old, and 5 months old devices, respectively (Figure S6B, Supporting Information). These correspond to 2%, 10%, and 17% change in responsivity for FHA proteins for 1, 2, and 5 month old devices, respectively, compared to the responsivity of the fresh devices. These results suggest that the high resistivity silicon devices preserve their performance at least for 2 months (responsivity change <10% with respect to the fresh devices) in detecting both RBD and FHA proteins.

The same experimental methodology was followed using devices fabricated on a relatively lower resistivity silicon wafer (1000–2000 Ω cm) to compare the results with the devices fabricated with a high resistivity silicon wafer. The devices fabricated on the lower resistivity silicon wafers also demonstrated a linear relationship in resistance change as a function of both RBD and FHA proteins (Figure S7, Supporting Information) with the same detection limit (7 pg mL^−1^). However, the responsivity for RBD and FHA proteins was determined to be 8.0 and 6.8 %/mg mL^−1^, respectively. These responsivity values are 9% and 13% lower for RBD and FHA proteins, respectively, compared to the corresponding responsivities of the devices on high resistivity silicon wafer. This suggests that high resistivity silicon wafers are more suitable in fabricating these biosensors for more sensitive biomolecule detection.

The plausible explanation for the resistance changes observed for the protein binding with COVID‐19 nanoMIPs is the intermolecular charge transfer supported by the intrinsic silicon wafer. Though the high resistivity intrinsic silicon wafer contains a relatively lower amount of charge carriers compared to the lower resistivity counterparts, these charge carriers are sufficient for electron transportation across the electrodes. The protein detection results from low resistive silicon wafers (Figure S7, Supporting Information) suggests that excessive charge carriers in silicon wafer do not necessarily improve the device sensitivity/detection limit/efficacy. The higher sensitivity observed for high resistive silicon wafers compared to its low resistive counterparts could possibly be due to the larger change in the electron flow with respect to the lower amount of charge carriers present in the high resistive silicon wafers upon interacting with proteins. The electron flow change with respect to the higher amount of charge carriers in low resistive silicon wafers is low upon interacting with proteins. Hence, a higher change in resistance was observed for high resistive silicon wafers than its low resistive counterparts for a given protein concentration leading to higher sensitivity. Increased resistance in the presence of proteins indicates that the incoming proteins act as electron acceptors, thus adopting electrons from the systems which ultimately decreases its conductivity.

**Figure 4:**
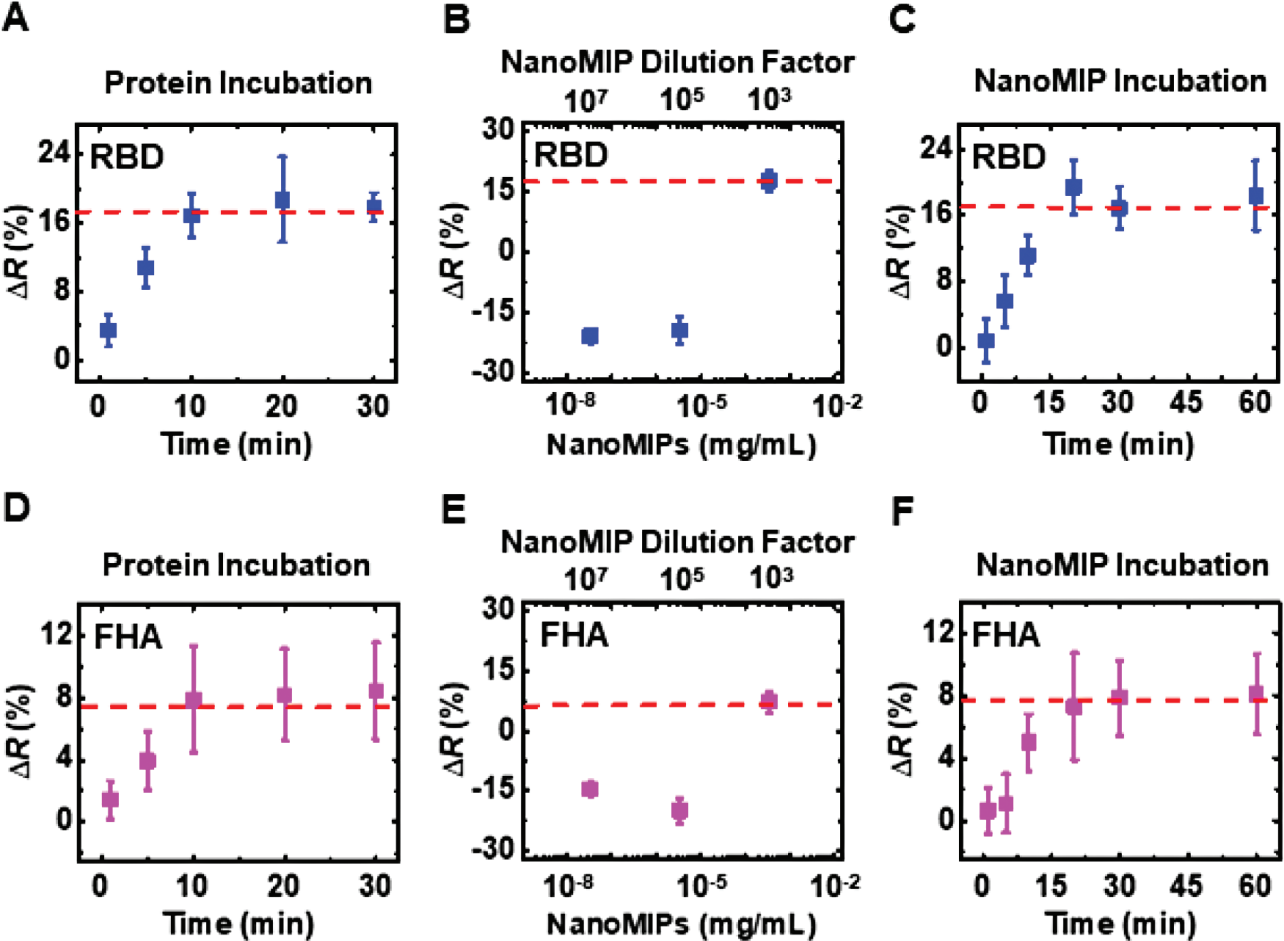
Effect of SARS‐CoV‐2 protein incubation time, nanoMIP concentration‐dependency, and nanoMIP incubation time. A) Change in resistance as a function of RBD protein incubation time. B) Change in resistance as a function of nanoMIP concentration for RBD protein detection. C) Change in resistance as a function of nanoMIP incubation time for RBD protein detection. D) Change in resistance as a function of FHA protein incubation time. E) Change in resistance as a function of nanoMIP concentration for FHA protein detection. F) Change in resistance as a function of nanoMIP incubation time for FHA protein detection. *n* = 5 for the statistical analysis of each data point.

Time‐dependent resistance change measurements of both RBD and FHA proteins suggest that the minimum time required for the maximum outcome for a given protein concentration is 10 min (**Figure** **4**A–D). The selected protein concentration for this study was 70 pg mL^−1^. Usually, the protein binding to corresponding natural antibodies is fast (<5 min).^[^ ^52^ ^]^ The COVID‐19 nanoMIPs are polymeric nanoparticles (≈70–150 nm diameter) which are several magnitudes higher in size compared to the natural antibodies (approximately several angstroms in size). Therefore, the packing density of COVID‐19 nanoMIPs is lower than that of natural antibodies and there is a certain degree of binding sites of COVID‐19 nanoMIPs that could be hindered during packing. Hence, it may take a longer incubation time for RBD and FHA proteins to successfully bind to the binding sites of the COVID‐19 nanoMIPs, unlike in natural antibodies where the binding sites are readily exposed to the antigens.

The lowest COVID‐19 nanoMIP concentration for both RBD and FHA protein detection is 0.33 µg mL^−1^ (corresponding to 1000‐fold dilution of the as‐received COVID‐19 nanoMIPs) (Figure 4B–E). Negative resistance changes were observed for the COVID‐19 nanoMIP concentrations lower than that of 0.33 µg mL^−1^. This could possibly be due to the direct interaction of RBD and FHA proteins and PBS ions with GPS molecules as the surface was not fully passivated with COVID‐19 nanoMIPs. The optimum incubation time for COVID‐19 nanoMIPs was determined to be 20 min for both RBD and FHA proteins (Figure 4C–F). This is three times faster than the initial measurements obtained for RBD and FHA protein detection where the incubation time of COVID‐19 nanoMIPs was 60 min. This result indicates that though the COVID‐19 nanoMIPs are polymeric nanoparticles with large molecular size, they can relatively quickly interact with GPS molecules.

### 2.4 Cross‐Selectivity Study on RBD and FHA Protein Detection

**Figure 5:**
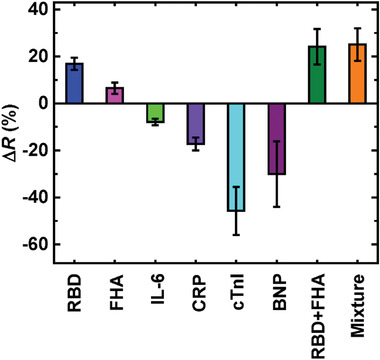
Cross‐selectivity studies for RBD and FHA detection. The resistance changes are shown for individual proteins used in the study, a solution with RBD and FHA proteins only, and a solution with a mixture of all the proteins used in the study. The nominal concentration of each protein is 70 pg mL^−1^. *n* = 5 for the statistical analysis of each data point.

The COVID‐19 nanoMIPs‐immobilized biosensors can selectively detect both RBD and FHA proteins in the presence of a mixture of other proteins (**Figure** **5**). This conclusion was drawn based on the experiment conducted for a mixture of proteins in the presence of both RBD and FHA proteins. Since both RBD and FHA proteins are available in SARS‐CoV‐2 virus particles, we first determined the resistance change for the RBD and FHA protein combined solution. The resistance change for the combined solution was calculated to be ≈24% which was approximately the same as of the additive resistance changes for the individual RBD (≈16%) and FHA (≈6%) protein samples. In contrast, other protein samples used for the selectivity study (i.e., interleukin‐6 (IL‐6), C‐reactive protein (CRP), cardiac Troponin I (cTnI), and brain natriuretic peptide (BNP)) displayed negative resistance changes on COVID‐19 nanoMIPs. The molecular sizes of these proteins [IL‐6 (≈21 kDa), CRP (≈25 kDa), cTnI (≈24 kDa), and BNP (≈3 kDa)] are relatively lower than that of RBD (≈30 kDa) and FHA (≈170–180 kDa) proteins. It is important to note that the resistance change observed for the mixture of all these proteins (RBD, FHA, IL‐6, CRP, cTnI, and BNP) is ≈26% which is close to the additive resistance changes of the individual RBD and FHA proteins. This suggests even the smaller proteins cannot directly bind to the COVID‐19 nanoMIPs highlighting that the COVID‐19 nanoMIPs are selective for RBD and FHA proteins.

### 2.5 Detection of SARS‐CoV‐2 Variant Proteins

**Figure 6:**
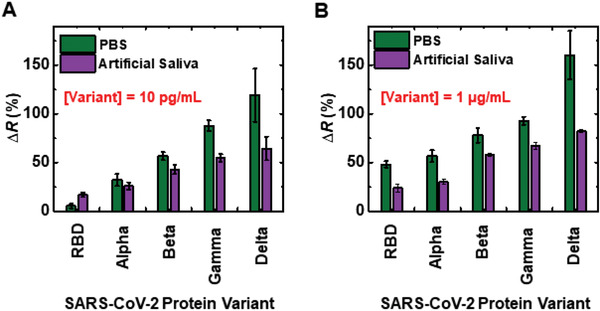
Detection of SARS‐CoV‐2 protein variants. A) Change in resistance for different SARS‐CoV‐2 protein variants at 10 pg mL^−1^ concentration. B) Change in resistance for different SARS‐CoV‐2 protein variants at 1 µg mL^−1^ concentration. *n* = 5 for the statistical analysis of each data point.

Our COVID‐19 biosensor can detect SARS‐CoV‐2 protein variants in both PBS and artificial saliva media (**Figure** **6**). Given the evolvement of the SARS‐CoV‐2 virus to different variants, it is important to develop methods to rapidly detect these variants as the severity of the infected COVID‐19 infection depends on the type of COVID‐19 variant.^[^ ^60^, ^61^ ^]^ Our biosensor can detect all four model SARS‐CoV‐2 protein variants used in the study (Alpha, Beta, Gamma, and Delta variants). To compare the SARS‐CoV‐2 protein variant responses with respect to the RBD reference strain response, commercially available RBD protein with a relatively similar amino acid sequence to the COVID‐19 variants was chosen for the study. The change in resistance for a given variant is increased with the variant concentration in both PBS and artificial saliva media. The lowest variant concentration tested was 10 pg mL^−1^ which is closer to the detection limit for RBD protein for our biosensor. The resistance change for the SARS‐CoV‐2 protein variants is in the order of RBD reference<Alpha<Beta<Gamma<Delta in both PBS and artificial saliva. The resistance changes for all SARS‐CoV‐2 protein variants for a given concentration are higher in PBS than in artificial saliva. The ionic strength of artificial saliva is much higher than PBS due to the high proportion of ionic components present in it. This is reflected in the resistance change observed for the control samples where artificial saliva had an average ‐50% resistance change compared to the average ‐12% resistance change in PBS. This strong ionic composition might have affected in charge transfer effect of SARS‐CoV‐2 protein variants upon interacting with COVID‐19 nanoMIPs, thus causing a lower resistance change compared to that of PBS for the same sample. A successful detection of COVID‐19 variants was also observed on low resistivity silicon devices (Figure S8, Supporting Information).

### 2.6 Reusability Study of the Biosensor

The capability of reusing our COVID‐19 biosensor was determined by treating the biosensor after the first usage with SARS‐CoV‐2 proteins under six different conditions: i) heating in pure water, ii) heating in PBS, iii) treating with acetic acid, iv) treating with urea, v) treating with Instrumax Pink disinfectant, and vi) treating with IPA. Heating treatments^[^ ^62^, ^63^ ^]^ and urea^[^ ^64^, ^65^ ^]^ were selected as both are well‐known protein denaturants to evaluate whether the denaturing of SARS‐CoV‐2 proteins could help to remove them from the nanoMIPs. Acetic acid and IPA were selected as the use of acids and alcohols to regenerate biosensors has been previously reported.^[^ ^66^ ^]^ Instrumax Pink disinfectant is a Therapeutic Goods Administration (TGA), Australia approved disinfectant in attenuating viruses including SARS‐CoV‐2. The efficiency of each treating method in reusing the biosensors was evaluated in two ways. First, the efficiency of removal of the SARS‐CoV‐2 proteins from the nanoMIPs without removing the nanoMIPs from the sensor. Second, the efficiency of rebinding the SARS‐CoV‐2 proteins onto the treated sensor in a second use cycle. Commercial RBD protein was used as the model SARS‐CoV‐2 protein in this study.

**Figure 7:**
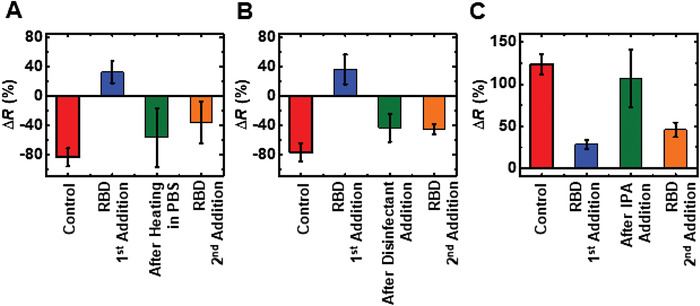
Reusability studies of SARS‐CoV‐2 biosensors. Evaluation of the efficiency of reusing the SARS‐CoV‐2 biosensor after A) heating in PBS, B) treating with Instrumax Pink disinfectant, and C) treating with IPA. *n* = 3 for the statistical analysis of each data point.

Successful removal of RBD protein from nanoMIPs was observed only for heating in PBS at 80 °C, treating with Instrumax Pink disinfectant, and treating with IPA (**Figure** **7**). This conclusion was derived based on the comparison of the resistance change behavior of the controls and the after the application of the treatments. If the resistance change for the controls and resistance change in the system after the treatment are in same polarity with comparable magnitude, that indicates the treatment can completely remove RBD proteins from the sensor. In contrast to these three treatments, acetic acid treatment could only partially remove RBD proteins whereas heating in water and treating in urea did not display any promising results for RBD removal at all (Figure S9, Supporting Information).

However, an appreciable amount of RBD protein binding to the pretreated sensor in the second RBD addition was only observed for IPA‐treated devices (Figure 7C). The plausible explanation for the failure in RBD second addition to the PBS heated sensors (Figure 7A) and Instrumax Pink disinfectant‐treated sensors (Figure 7B) could possibly be due to any structural modifications of the nanoMIPs and/or the silicon device itself occurring due to interaction with the disinfectant. Being a less strong chemical and high volatility, IPA might have the least damage to the nanoMIP‐immobilized sensor surface. Therefore, the incoming RBD in the second addition following IPA treatment is quite successful in binding to the retained nanoMIPs on the sensor surface.

### 2.7 Battery‐Free, Wireless Device Design and Its Performance

**Figure 8:**
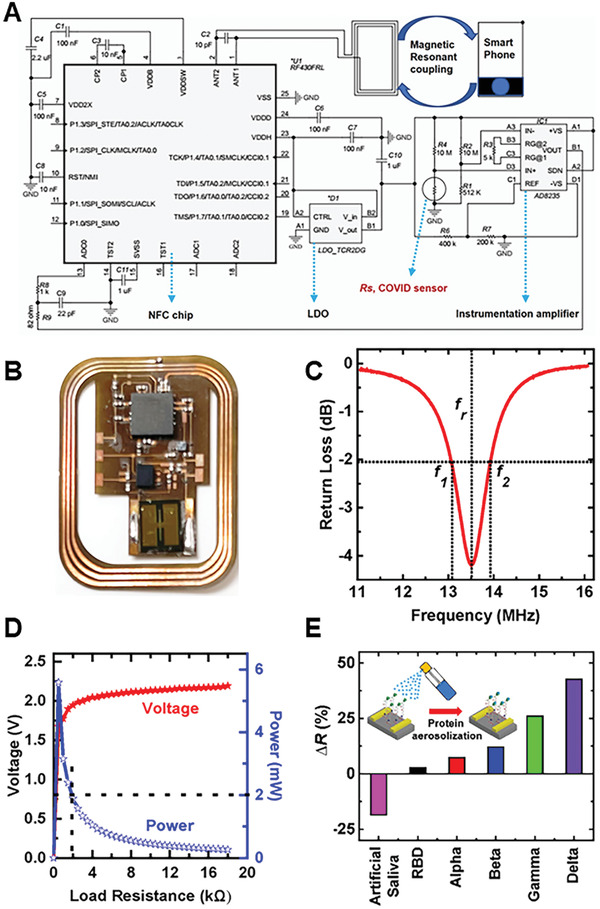
Touchless, instant detection of SARS‐CoV‐2 protein variants. A) Schematic design of battery‐free wireless circuit, B) Fabricated device, C) Q‐factor and resonance frequency matching at 13.56 MHz. D) Energy harvesting to ensure the device operation, and E) SARS‐CoV‐2 protein variant detection by using the spraying method according to the resistance change.

To wirelessly transmit instant sensing response to the cloud connected interface, a battery‐free near‐field communication (NFC) system was employed. The wireless electronic measurement device was powered through a magnetically coupled primary coil of the NFC supported smartphone and a secondary loop coil incorporated on the device resonating at 13.56 MHz. The induced voltage in the secondary loop coil was fed into the half‐wave bridge rectifier followed by a low‐dropout voltage regulator (1.8 V) to enable a constant and uninterrupted dc power supply to the circuit. **Figure** **8**A illustrates the basic operational principle of the entire circuit. The analog front‐end comprised of a Wheatstone bridge with resistances R1, R2, Rs, and R4 to detect any resistance change of the sensor, Rs, due to incoming covid variants and a differential amplifier configuration to distinguish and amplify the voltage difference occurs because of resistance changes of the sensor. The analog‐to‐digital converter (ADC0) of NFC SoC (System on Chip) digitalized the incoming signal from the amplifier output pin (B1) and the system transmitted the digitalized data over air using ISO/IEC 15693 protocol for contactless data reading by smartphone. An android app (FMM Connect as demonstrated in Video S1, Supporting Information) was developed to receive and plot the data from the NFC SoC.

Figure 8B represents the fabricated device where the circuit board was printed using a laser micromachining, and the components with a form factor of 0201 were attached on the board exploiting a reflow‐soldering machine. The detailed fabrication process was described in our recent publication.^[^ ^67^ ^]^ The performances of secondary loop coil antenna and energy harvesting by the antenna to power the active and passive components were the key factors for reliable wireless data transmission. To achieve the resonant frequency of 13.56 MHz, a capacitor of 10 pF was utilized between the pins of 1 and 2 of NFC SoC. Figure 8C illustrates the resonance peak position (*f*_r_) at 13.56 MHz of the loop coil antenna measured by a precision spectrum analyzer. The narrow bandwidth (*f*_2_ ‐ *f*_1_) of full width half maximum (FWHM) demonstrated higher Q‐factor (*f*_r_/(*f*_2_ ‐ *f*_1_)) indicating low rate of energy loss of loop coil antenna.

Figure 8D illustrates the unregulated output voltage and corresponding power across a range of load resistor. While the maximum power of 5.5 mW was harvested at 500 Ω, the device load resistance shows a power of 2 mW which was sufficient for the peak power consumption of NFC SoC and analog frontend according to their data sheets RF430FRL152H and AD8235.

To evaluate the efficacy of the SARS‐CoV‐2 biosensor in detecting the SARS‐CoV‐2 proteins to the end‐user using the fabricated device, an aerosolized protein experiment was conducted to mimic the saliva and mucosa droplets that are released during sneezing (Figure 8E). A commercial spray bottle was used in the experiment to spray the proteins onto the nanoMIP‐immobilized sensors. The details of the experiment can be found under Experimental Section. Similar to the observation in the drop casted method (Figure 6), the aerosolized/spray SARS‐CoV‐2 protein on sensor attached to the wireless device displayed a positive change in resistance in the same order of Delta>Gamma>Beta>Alpha>RBD reference. The control of solvent artificial saliva produced a negative contribution to the resistance change. In order to calculate the resistance change of the sensor, the hexadecimal data received from the smartphone was converted to the decimal input to the ADC0 by using the following equation according to the NFC SoC datasheet:

(2) $$\begin{array}{c} \text{ADC}\;\text{input}=\frac{\text{Hexadecimal}\;\text{value}}{2^{14}-1}\times 0.9\;V \end{array}$$

Then the output of the instrumentation amplifier is calculated by subtracting the reference voltage set up for the amplifier according to Equation 2.

(3) $$\begin{array}{c} \text{Output}\;\text{of}\;\text{amplifier}=\left(\text{ADC}\;\text{input}-0.45\right)V \end{array}$$

Without employing a gain‐setting resistance marked as R3 between the pins B3 and C3 of the amplifier, the amplifier gain is 5 according to the datasheet of AD8235. Hence, the sensor resistance (Rs) can be calculated by using the following equation due to the Wheatstone bridge.

(4) $$\begin{array}{c} \left(\frac{\text{Rs}}{\left(R4+\text{Rs}\right)}-\frac{R1}{\left(R1+R2\right)}\right)1.8=\frac{\text{Output}\;\text{of}\;\text{amplifier}}{5} \end{array}$$

where, R1, R2, and R4 are 512 kΩ, 10 MΩ, and 10 MΩ, respectively.

The equations are incorporated in the android app to display the resistance change, Δ*R*(%), and to determine the corresponding COVID variants. Figure 8E illustrates the detection of covid variants which agrees to the drop casted detection of COVID variants as demonstrated in Figure 6.

## 3 Discussion

A highly selective and sensitive conductometric biosensor for the rapid detection of SARS‐CoV‐2 proteins and its variants was developed in the present study. The sensor platform consisted of a commercial high resistivity silicon wafer and photolithographically developed two‐terminal electrodes. After surface functionalization with GPS silanization, COVID‐19 nanoMIPs were immobilized for selective binding of SARS‐CoV‐2 surface proteins. Both model SARS‐CoV‐2 proteins; RBD and FHA, displayed a linear relationship in change in resistance with the protein concentration with both displaying a detection limit of 7 pg mL^−1^. The sample immobilization time was as low as 10 min and the readings were obtained in <1 min. The shelf‐life of the biosensor was determined to be about 2 months. This biosensor was extremely selective to the detection of model SARS‐CoV‐2 proteins even in the presence of other types of proteins with similar molecular sizes used in the study. In addition, this biosensor could detect different variants of SARS‐CoV‐2 proteins in the order of resistance changes at Delta>Gamma>Beta>Alpha>RBD reference. Another important finding in this study was that pure IPA and acetic acid can be used for the complete and partial removal of SARS‐CoV‐2 proteins from the sensor surface, respectively, without detrimental damage to the nanoMIPs or sensor integrity. Hence, the sensor can be used at least for two cycles of RBD protein detection when regenerated with these treatments. By using NFC circuitry, the SARS‐CoV‐2 protein detection results were successfully transferred to a mobile app. The aerosolized protein experiment which mimics the release of saliva and mucosa droplets during sneezing also displayed the resistance changes for the SARS‐CoV‐2 protein variants in the order of Delta>Gamma>Beta>Alpha>RBD reference. Collectively, this study demonstrates the power of using a high resistivity silicon wafer‐based conductometric sensor platform for the development of an economically viable, sensitive, and reusable next‐generation point‐of‐care medical device.

## 4 Experimental Section

### Device Fabrication

Silicon wafers (resistivities: >5000 Ω cm and 1000–2000 Ω cm) for biosensor fabrication were purchased from D&X Co. Ltd. (Japan). As‐received Si wafers were rinsed sequentially in acetone, IPA, and deionized (DI) water followed by drying under compressed N_2_ gas. These Si wafers were then dehydrated at 120 °C for 2 min. AZ 5214E photoresist was spin coated on the dehydrated Si wafers using a SPIN150 spin coater for 30 s at a spinning speed of 3000 rpm. The Si wafers were then soft baked at 95 °C for 1 min. The electrode patterns with desired electrode gaps and lengths were designed on the Si substrate using a MLA150 Maskless Aligner (Heidelberg Instruments). The electrode patterns were then hard baked at 120 °C for 2 min followed by flood exposure for UV radiation for 15 s. Finally, the substrate was developed in a 400 K developer for 20 s followed by 30 s of rinsing in DI water. The electrodes were then tested under a light microscope to ensure there were no remaining photoresists between the electrode gaps. The electron beam deposition (PVD75 E‐beam Evaporator, Kurt J. Lesker) was used to deposit a 30 nm titanium adhesion layer and a 300 nm gold electrode layer. At the end of the photolithography process, the electrode patterns were realized by a lift‐off process.

### GPS Silanization

GPS silane was purchased from Sigma‐Aldrich (Castle Hill, New South Wales, Australia) and used as received. GPS silanization was conducted by the same procedure followed according to our recent publication.^[^ ^52^ ^]^ In brief, freshly prepared Si devices were exposed to O_2_ plasma for 10 min (Plasma Cleaner PDC‐002, Harrick Plasma). These devices were then exposed to GPS vapor for 1–2 h in a vacuum desiccator placed inside a fume hood. Then, the devices were rinsed thoroughly with DI water for 2 min followed by heating at 150 °C for 10 min.

### Surface Characterizations

FTIR spectra of the samples were acquired using a Spotlight 400 FT‐IR Imaging System equipped with Perkin Elmer Spectrum and Spectrum IMAGE Viewer software. The size of the scanned area was 100 µm × 100 µm and an average of 516 scans were taken for a given scanned spot. Three spots were scanned for a given sample and the average spectra of the sample were reported in the manuscript. Water contact angle measurements were obtained using a Dataphysics OCA 15 plus Contact Angle instrument equipped with SCA 20 software.

### Details of SARS CoV‐2 S2P_1208_‐FHA Protein

SARS CoV‐2 S2P_1208_‐FHA protein was provided by the Burnet Institute, Melbourne, Australia. This protein comprised a tissue plasminogen activator leader sequenced linked through Ala‐Ser to residues 16‐1208 of the Wuhan Hu‐1 (Genbank accession number: YP_009724390.1) S ectodomain. The protein includes the modifications, K986P/V987P (to maintain prefusion conformation), R628ARR→G628SAS mutation (to remove the furin cleavage site),^[^ ^68^ ^]^ and addition of GlySerGlySer‐foldon‐GSGS‐His8‐GSGS‐Avitag (GLNDIFEAQKIEWHE) sequence to the C‐terminus.

The glycoprotein was expressed in 293F cells and purified from the supernatant via Co^2+^ TALON affinity chromatography followed by Superose 6 size exclusion chromatography (SEC). The purified glycoprotein appeared as a single symmetrical peak in SEC and a single band (170–180 kDa) in an SDS‐PAGE. Binding of S2P_1208‐_FHA protein to human ACE2‐Fc was confirmed in biolayer interferometry. The sample had been filter‐sterilized and contained 200 µg S2P_1208_‐FHA (290 µL @ 0.7 mg mL^−1^) in PBS. The concentration was inferred from the absorbance at 280 nm (extinction coefficient = 1.019).

### Details of SARS CoV‐2 RBD of Spike Glycoprotein

SARS CoV‐2 RBD of spike glycoprotein was provided by the Burnet Institute, Melbourne, Australia. This protein comprised the RBD ectodomain sequence (amino acids 332‐532) from the Wuhan Hu‐1 isolate (Genbank accession number: YP_009724390.1) linked to the TPA leader sequence. Four key modifications were included: i) A serine at the first position for optimized leader sequence cleavage, ii) GGSGS flexible linker after final ^532^N, iii) HHHHHHHH poly His tag for purification and detection with anti‐HIS antibodies, and iv) Avi tag for site‐specific biotinylation.

The glycoprotein was expressed in 293 expi cells and purified from the supernatant via Co^2+^ TALON affinity chromatography followed by Superdex200 16/600 size exclusion chromatography (SEC). The purified glycoprotein appeared as a single symmetrical peak in SEC and a single band (30 kDa) in an SDS‐PAGE. Binding of the RBD to human ACE2‐Fc was confirmed in biolayer interferometry. The sample was filter‐sterilized and contained 100 µg of protein at 2.14 mg mL^−1^. The concentration was inferred from the absorbance at 280 nm (extinction coefficient 1.48. OD280 of 1:10 = 0.317).

### Details of Commercial SARS CoV‐2 Protein Variants

SARS CoV‐2 spike protein (S‐RBD) (aa 330‐524), His tag recombinant protein (Catalogue No. RP87674) was purchased from Thermo Fisher Scientific (Scoresby, Australia). All SARS‐CoV‐2 protein variants (Alpha strain: SARS‐CoV‐2 (2019‐nCoV) Spike RBD (N501Y)‐His Recombinant Protein [Catalogue No. 40592‐V08H82], >95% purity; Beta strain: SARS‐CoV‐2 (2019‐nCoV) Spike RBD (K417N, E484K, N501Y)‐His Recombinant Protein [Catalogue No. 40592‐V08H85], >90% purity; Gamma strain: SARS‐CoV‐2 (2019‐nCoV) Spike RBD (K417T, E484K, N501Y) Protein (His Tag) [Catalogue No. 40592‐V08H86], >95% purity; and Delta strain: SARS‐CoV‐2 Spike RBD (T478K) Protein (His Tag) [Catalogue No. 40592‐V08H91]) were purchased from Sino Biological Inc. (China).

### SARS CoV‐2 nanoMIPs, Antigen Solutions, and Other Chemicals

SARS‐CoV‐2 nanoMIPs solution (with 0.05% v/v ProClin 300 preservative, concentration of as‐received nanoMIPs was 0.339 mg mL^−1^, particle size ranging from ≈70–150 nm) was provided by MIP Discovery Ltd. (UK). IL‐6, CRP, and 1× PBS (pH 7.4) solutions were purchased from Sigma‐Aldrich (Castle Hill, New South Wales, Australia). Human cTnI antigen was purchased from HyTest Ltd. (Turku, Finland). Human BNP antigen was purchased from Bachem (Bubendorf, Switzerland). Artificial saliva was purchased from Pickering Laboratories Inc. (Mountain View, California, USA). IPA (purity >99.5%) was purchased from Merck KGaA (Germany). Urea (purity >99%) was purchased from Chem Supply (Australia) and was dissolved in PBS to produce a 10 m solution. Acetic acid (purity >99%) was purchased from Sigma‐Aldrich (Castle Hill, New South Wales, Australia). Instrumax Pink (Whiteley) disinfectant was purchased from Total Cleaning (Melbourne, Australia). All materials were used as received.

### Immobilization of SARS‐CoV‐2 NanoMIPs

SARS‐CoV‐2 nanoMIP solution (10 µL) was drop cast onto freshly prepared GPS‐silanized Si devices and incubated for 0.5–1 h, allowing maximal binding of the nanoMIPs onto the devices. Then, the devices were rinsed extensively with PBS solution to remove any unbound nanoMIPs followed by drying with compressed N_2_ gas (>99.9% purity). The resistance across the nanoMIP‐immobilized electrodes (*R*_0_) was measured using a LTS120 Linkam Stage and a B2901A Precision Source/Measure Unit (Keysight Technologies). Keysight Quick I‐V Measurement software was used in acquiring resistance data.

### Immobilization of SARS‐CoV‐2 Proteins

All the as‐received SARS‐CoV‐2 protein solutions were diluted to the predetermined concentrations in either PBS or artificial saliva (depending on the experiment of interest). SARS‐CoV‐2 protein solution (10 µL) was drop cast onto the nanoMIP‐immobilized Si devices and incubated for 10 min. After 10 min, the remaining SARS‐CoV‐2 protein solution was pipetted out and the devices were dried under compressed N_2_ gas (>99.9% purity). The resistance across the electrodes was measured using a LTS120 Linkam Stage and a B2901A Precision Source/Measure Unit (Keysight Technologies). Keysight Quick I‐V Measurement software was used in acquiring resistance data. In each experiment, 3‐5 devices were used, and the average values were utilized in the change in resistance calculations.

### Statistical Analysis

For each calculated data point (∆*R*%) in the graphical representations of the experimental studies, 3‐5 devices were utilized (i.e., sample size (*n*) = 3 or 5). Prior to calculating the change in resistance (∆*R*%), the average resistance values for baseline measurements (*R*_0_) and average the resistance values after the antigen/protein addition (*R*) were evaluated for each individual sensor. The average resistance values (i.e., *R*_0_ and *R*) were evaluated by calculating the average resistance value monitored in each reading from 40 to 60 s range where a stable resistance signal was observed. The mean value for each data point (∆*R*%) in the graphs was calculated from averaging the ∆*R*% value calculated for each individual sensor (*n* = 3 or 5). The standard deviation (S.D.) was also evaluated for each data point from 3 to 5 individual ∆*R*% values. The error bar in each data point represents the standard error (S.E.) and it was calculated by using the formula; S.E. = S.D./√*n*. The standard Microsoft Office Excel spreadsheets were used in the statistical analysis. As the current research highlights the proof‐of‐concept study in detecting the COVID‐19 proteins and its variants by using our conductometric biosensors, further clinical evaluation is required for full statistical analysis.

## Conflict of Interest

The authors declare no conflict of interest.

## Supporting information

Supporting Information

Supplemental Video 1

## Data Availability

Research data are not shared.
