# Supplementary material for: Rapid Conductometric Detection of SARS‐CoV‐2 Proteins and Its Variants Using Molecularly Imprinted Polymer Nanoparticles
Source: Adv Mater Technol. 2022 Dec 4:2200965. Online ahead of print. doi: 10.1002/admt.202200965 (PMC9877662; doi:10.1002/admt.202200965)
Supplement: Supplementary file 1 — Supporting Information [file ADMT-9999-0-s002.pdf]

ADVANCED MATERIALS TECHNOLOGIES

Supporting Information

for *Adv. Mater. Technol.,* DOI: 10.1002/admt.202200965

Rapid Conductometric Detection of SARS-CoV-2 Proteins and Its Variants Using Molecularly Imprinted Polymer Nanoparticles

Ganganath S. Perera, Md. Ataur Rahman,* April Blazevski, Alasdair Wood, Sumeet Walia, Madhu Bhaskaran, and Sharath Sriram**

Supporting Information

Rapid conductometric detection of SARS-CoV-2 proteins and its variants using molecularly imprinted polymer nanoparticles

Ganganath S. Perera,[1]* Md. Ataur Rahman,[1] April Blazevski,[1] Alasdair Wood,[2] Sumeet Walia,[1] Madhu Bhaskaran,[1] and Sharath Sriram[1]*

[1] Functional Materials and Microsystems Research Group and the Micro Nano Research Facility, RMIT University, Melbourne, Australia

[2] Soterius Pty Ltd., Melbourne, Australia

*Corresponding authors' email: ganganath.perera@rmit.edu.au, mdataur.rahman@rmit.edu.au, and sharath.sriram@rmit.edu.au

Content **Page No.**

S1. Optimization of Electrode Gaps and Electrode Lengths for Devices S3

S2. RBD Protein Detection at Different Electrode Lengths of Devices S3

S3. Resistance Measurements After GPS Silanisation on High Resistivity Silicon Devices S4

S4. FTIR Spectra and Resistance Measurements After NanoMIP and SARS-CoV-2 Protein Binding S4

S5. FTIR Spectra After SARS-CoV-2 Proteins are Bound to NanoMIPs S5

S6. Shelf-life Evaluation of the GPS Silanised High Resistivity Silicon Devices on RBD and FHA Protein Detection S5

S7. RBD and FHA Protein Detection on Low Resistivity Silicon Devices S6

S8. Detection of COVID-19 Protein Variants on Low Resistivity Silicon Devices S6

S9. Reusability Study for SARS-CoV-2 Biosensor using Water Heating, Acetic Acid and Urea Treatments S7

S1. Optimization of Electrode Gaps and Electrode Lengths for Devices

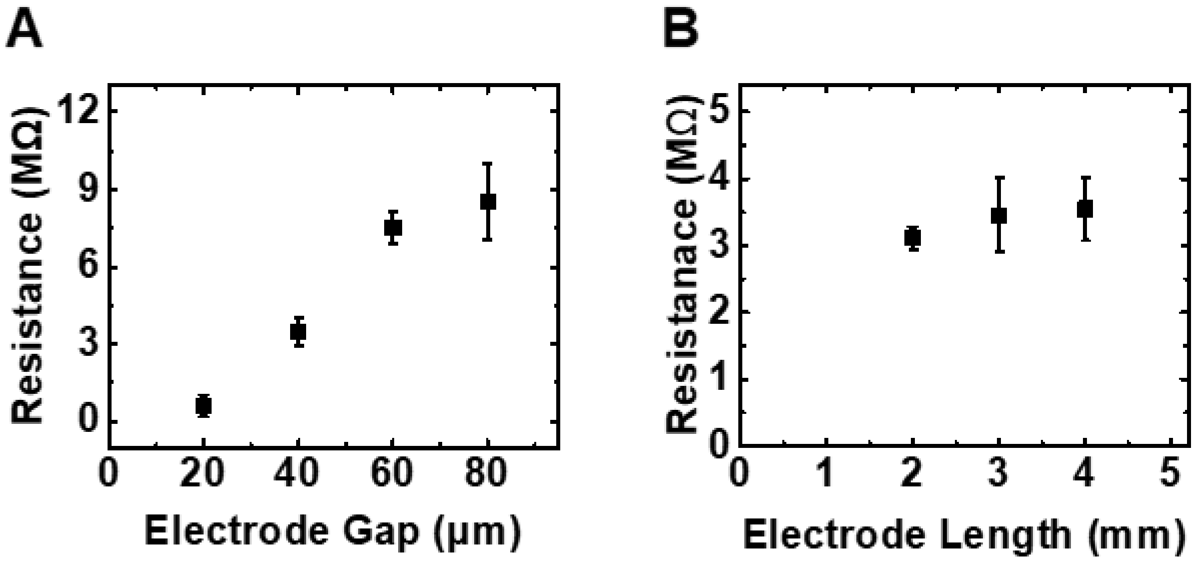

Figure S1 | Optimization of electrode gaps and electrode length. (A) Resistance across the electrodes as a function of gap between an electrode pair. (B) Resistance across the electrodes as a function of electrode length.

S2. RBD Protein Detection at Different Electrode Lengths of Devices

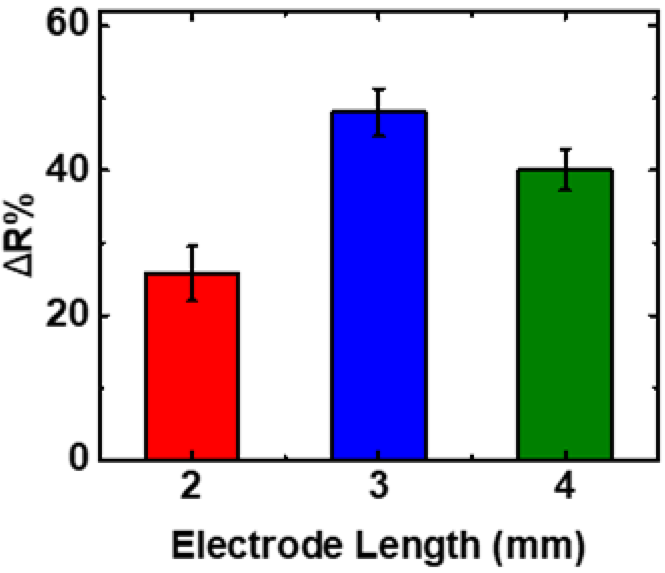

Figure S2 | RBD protein detection as a function of electrode length. (A) Change in resistance for RBD protein detection at different electrode lengths. The nominal RBD protein concentration is 0.7 mg/mL.

S3. Resistance Measurements After GPS Silanisation on High Resistivity Silicon Devices

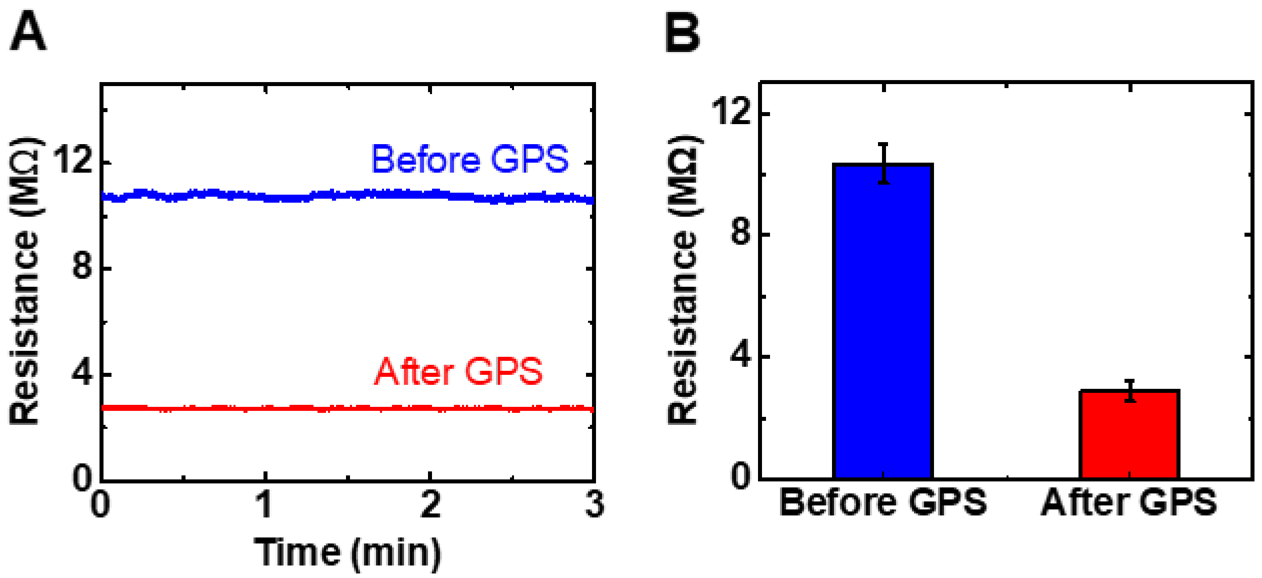

Figure S3 | Comparison of resistance values of the devices before and after GPS silanisation. (A) Variation of the resistance of the high resistivity Si devices before and after GPS silanisation as a function of time. (B) Calculated average resistance values of the high resistivity devices before and after GPS silanisation. The average values were calculated based on 5 devices each.

S4. FTIR Spectra and Resistance Measurements After NanoMIP Binding to GPS-Silanised Devices

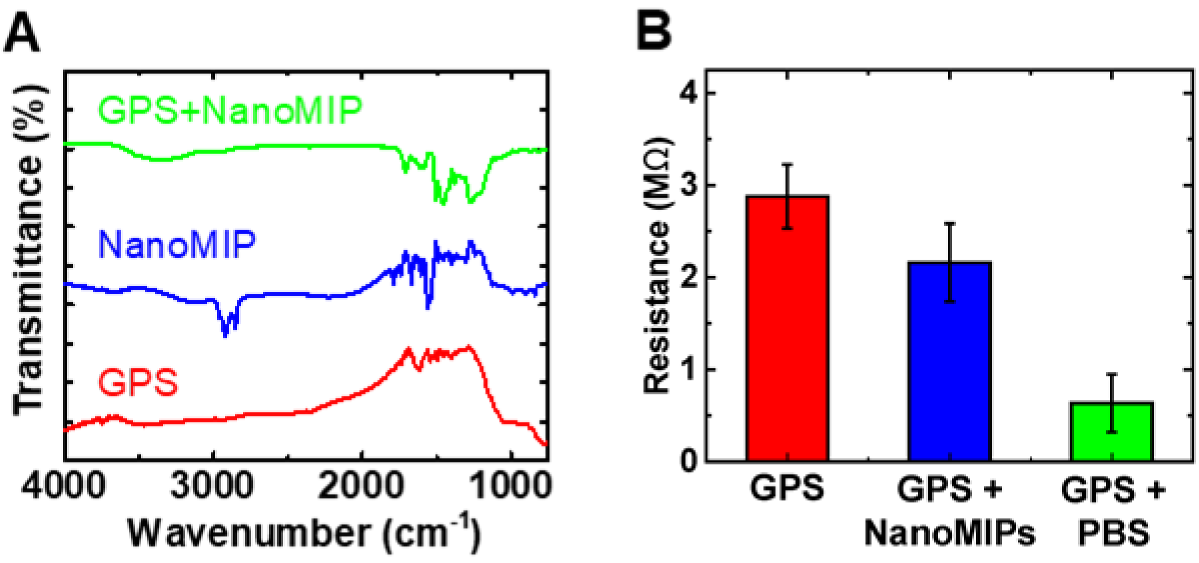

Figure S4 | FTIR spectra and resistance measurements for nanoMIP bound sensors. (A) FTIR spectra for the as-fabricated silicon devices with GPS only, nanoMIPs only, and GPS+nanoMIPs. (B) Resistance measurements for GPS silanised devices with nanoMIPs and PBS. The average values for each condition were calculated based on 5 devices each.

S5. FTIR Spectra After SARS-CoV-2 Proteins are Bound to NanoMIPs

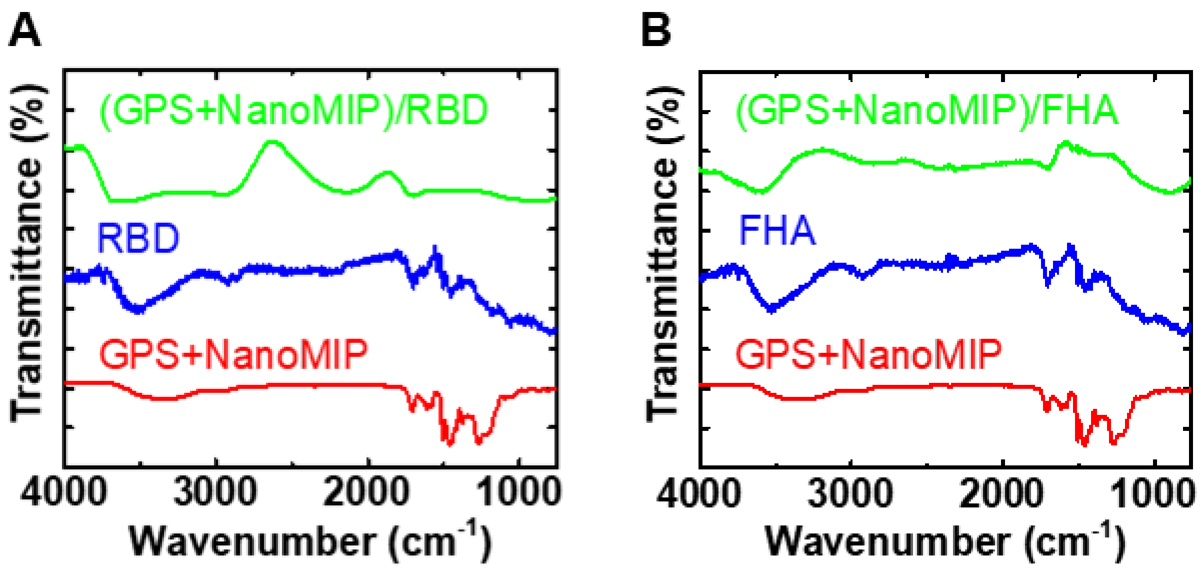

Figure S5 | FTIR spectra of RBD and FHA proteins for nanoMIP bound sensors. (A) FTIR spectra for the nanoMIPs on GPS silanised device, RBD only, and (GPS+nanoMIPs)+RBD. (B) FTIR spectra for the nanoMIPs on GPS silanised device, FHA only, and (GPS+nanoMIPs)+FHA.

S6. Shelf-life Evaluation of the GPS Silanised-High Resistivity Silicon Devices on RBD and FHA Protein Detection

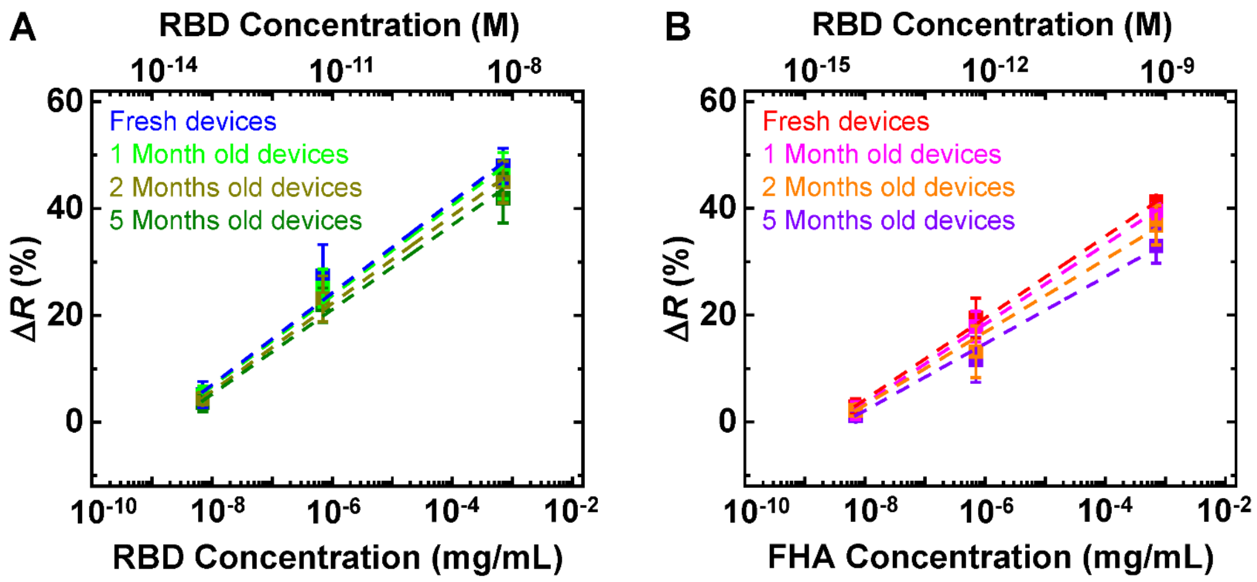

Figure S6 | Shelf-life evaluation of GPS silanised high resistivity silicon devices. Change in resistance values as a function of (A) RBD and (B) FHA protein concentrations on high resistivity silicon devices which GPS silanised at different timescales.

S7. RBD and FHA Protein Detection on Low Resistivity Silicon Devices

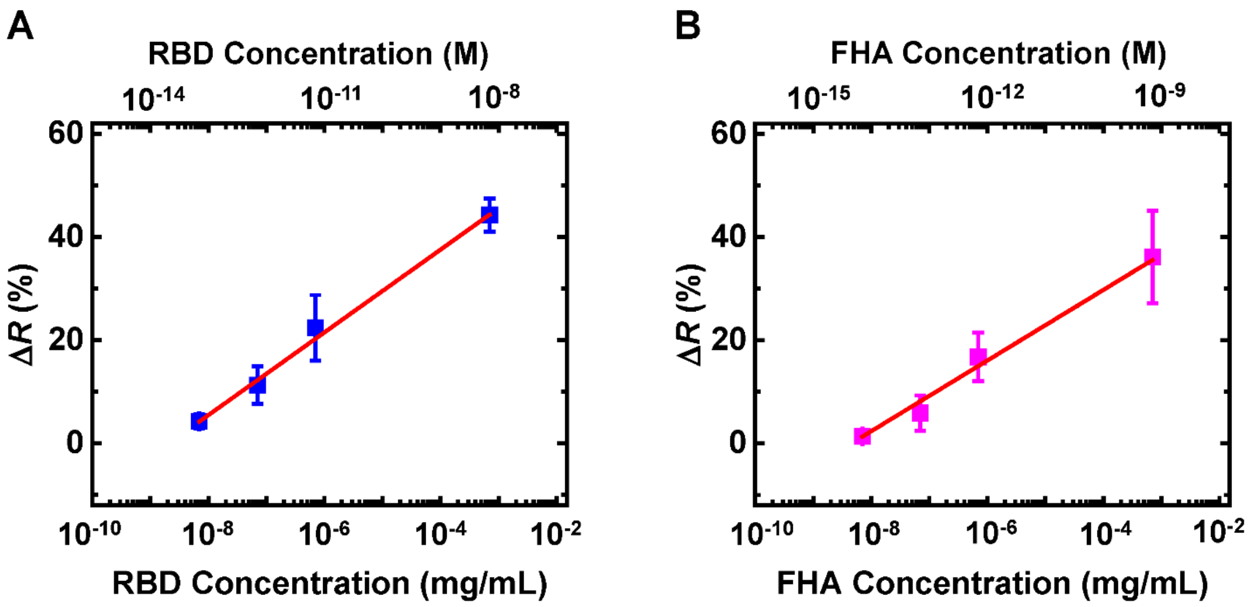

Figure S7 | Detection of model SARS-CoV-2 proteins on low resistivity silicon devices. Change in resistance values as a function of (A) RBD and (B) FHA protein concentrations on low resistivity silicon devices.

S8. Detection of COVID-19 Protein Variants on Low Resistivity Silicon Devices

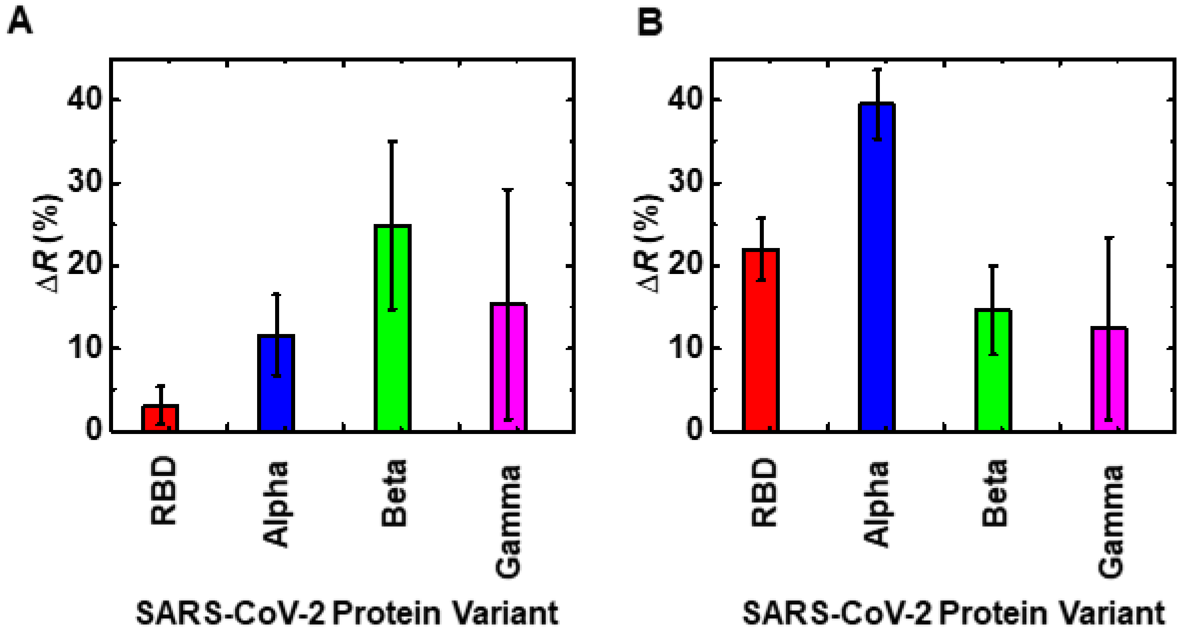

Figure S8 | Detection of COVID-19 protein variants on low resistivity silicon devices. Change in resistance values for different COVID-19 protein variants low resistivity silicon devices in PBS at nominal protein concentrations of (A) 10 pg/mL and (B) 1 µg/mL.

Low resistivity silicon devices also displayed successful detection of COVID-19 variants (Figure S8, Supporting Information). However, unlike the high resistivity silicon devices, the low resistivity silicon devices did not show a clear trend in detecting the SARS-CoV-2 protein variants as a function of protein concentration in PBS. The exact reason for this behaviour is currently unclear. This indicates that high resistivity silicon devices are much better performers in detecting SARS-CoV-2 protein variants than the low resistivity silicon devices.

S9. Reusability Study for SARS-CoV-2 Biosensor using Water Heating, Acetic Acid and Urea Treatments

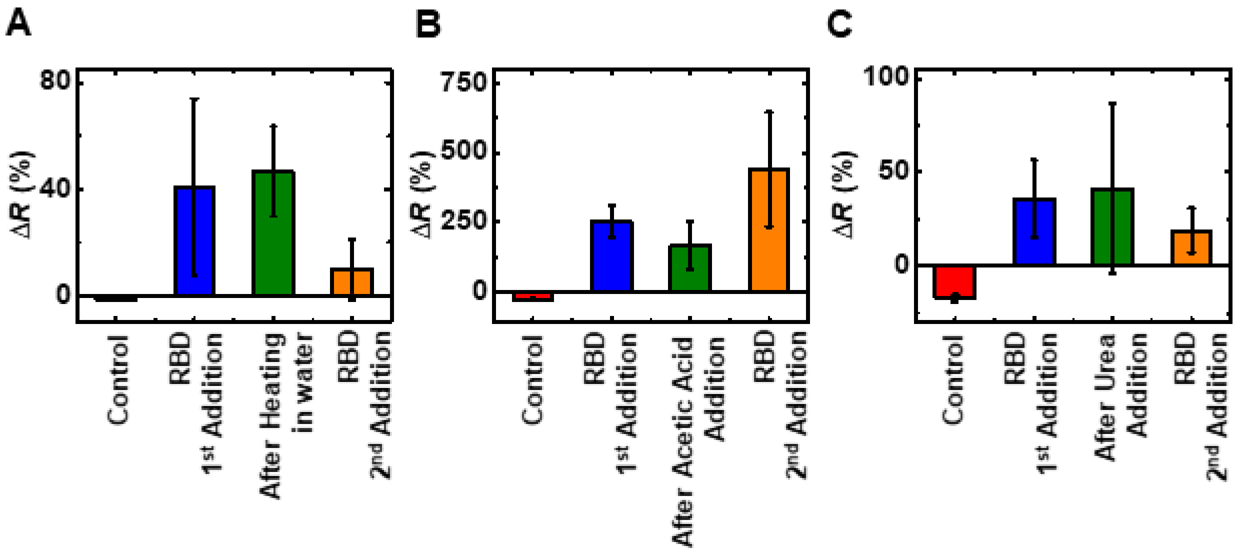

Figure S9 | Reusability study of SARS-CoV-2 biosensor at different conditions. Evaluation of reusability of SARS-CoV-2 biosensor at (A) heating in water, (B) acetic acid treatment, and (C) urea treatment.
